# Dyspnea, anxiety, and death anxiety in patients with COPD

**DOI:** 10.1017/S1478951525100382

**Published:** 2025-08-25

**Authors:** Gamze Yeğin, Songül Karadağ

**Affiliations:** Seyhan State Hospital, Adana, Türkiye; Department of Nursing, Cukurova University, Adana, Türkiye

**Keywords:** Chronic obstructive pulmonary disease, dyspnea, nursing, anxiety, death anxiety

## Abstract

**Objective:**

This study was conducted to identify dyspnea, anxiety, and death anxiety in patients with Chronic Obstructive Pulmonary Disease (COPD).

**Method:**

The study was carried out with 200 COPD patients who applied to the chest diseases outpatient clinic of a state hospital between December 2022 and June 2023.

**Results:**

A total of 73.0% of the patients with COPD participating in the study were male and their mean age was 66.73 ± 8.45 years. Their mean scores were 5.21 ± 2.46 on Modified Borg Scale, 2.62 ± 1.03 on the Modified Medical Research Council scale, 17.87 ± 7.96 on the Beck Anxiety Inventory, and 10.07 ± 4.02 on the Death Anxiety Scale. Patients with high dyspnea levels also had high levels of anxiety and death anxiety (*p* < 0.001).

**Significance of results:**

The patients with COPD had high levels of dyspnea, anxiety, and death anxiety. Based on the results of the study, it is recommended to plan evidence-based studies to alleviate dyspnea, anxiety, and death anxiety in patients with COPD.

## Introduction

Chronic obstructive pulmonary disease (COPD) is a condition characterized by chronic respiratory symptoms caused by airway or alveolar abnormalities along with persistent and often progressive airway obstruction (Global Initiative for Chronic Obstructive Lung Disease (GOLD) 2024). COPD ranks the third leading cause of death worldwide, accounting for approximately 3 million deaths annually (World Health Organization, 2023). According to the Global Initiative for Chronic Obstructive Lung Disease (GOLD) 2024 report, COPD-related deaths are estimated to be more than 5.4 million by 2060 (GOLD 2024). According to Turkish Statistical Institute (TURKSTAT)’ 2022 data, respiratory system diseases (13.5%) rank the third leading cause of death in Türkiye after circulatory system diseases (35.4%) and cancers (15.2%) (TURKSTAT 2022). COPD and bronchiectasis-related deaths account for 5.4% of all deaths (TURKSTAT 2019). The GOLD’s 2024 report indicates that the global prevalence of COPD is 10.3% (GOLD 2024). The prevalence of COPD over 40 years of age was reported to be 19.1% in the BOLD-Turkey study (Kocabaş et al. 2006).

COPD affects adversely the lives of patients physically, emotionally, cognitively, socially, and economically and limits their activities of daily living (Durna 2012). The most common symptom of COPD is dyspnea that consists of sensual and emotional components. Typically, COPD patients describe dyspnea as an increased effort to breathe, chest tightness, or insufficient air intake or breathiness. However, the terms used to describe dyspnea may vary both individually and culturally (GOLD 2024). In their study, Carette et al. found that 53% of patients with COPD suffered from severe dyspnea despite inhaled drug treatments (Carette et al. 2019).

The sense of discomfort induced by dyspnea affects self-care skills of patients with COPD, so they have difficulty in their activities of daily living. This leads their functionality to diminish and causes them to experience anxiety and death anxiety (Turhal and Koç 2021). Anxiety is defined as one’s feeling of concern about a subjective condition that is unclear how it will take place or is unlikely to take place at all (Şahin 2019). The prevalence of anxiety in ranges between 10% and 55% in patients with COPD and between 13% and 46% in outpatients with COPD (Henning et al. 2020).

People feels anxiety due to unknown nature of death from chronic diseases such as COPD (Cengiz et al. 2021). Death anxiety refers to a conscious or unconscious psychological state resulting from a defense mechanism that can be triggered when patients face the danger of death and it develops after they become aware that they will no longer exist (Korkmaz and Tel 2010). In their study, Nal et al. reported that 75% of patients with COPD had death anxiety (Nal et al. 2016). Togluk and Çuhadar conducted a study with 150 COPD patients and found that these patients had a moderate level of death anxiety, and their social adaptation got impaired as their death anxiety increased (Togluk and Çuhadar 2021).

It is crucial to assess dyspnea, death anxiety, and anxiety in patients with COPD in order to enhance their mental well-being and quality of life (Nal et al. 2016). Dyspnea increases anxiety in patients. The resulting feeling of breathlessness causes death anxiety, which negatively affects patients both physically and psychologically. Individuals with high dyspnea perception are also at high risk for anxiety and death anxiety. Dyspnea signals an existential threat, and therefore appropriately provokes anxiety, motivating behaviors to escape the threat. Conversely, anxiety can increase or even generate dyspnea. Clark’s Cognitive Model of Panic Attacks (Clark 1986) suggests that panic attacks occur as a result of a specific sequence of events. These events are usually sensations felt in the body or mental experiences. According to the model, triggering stimuli can be external but are mostly internal (a bodily sensation, thought, or image) (Aslan 2025). When these stimuli are perceived as a threat by the individual, anxiety arises. The person interprets certain bodily sensations as a sign of an impending catastrophe (McNally 2001). When we examine our study within the framework of this model, the patient’s belief that they will die as a result of experiencing severe dyspnea attacks leads to death anxiety. The association of dyspnea with death in the patient’s internal world causes anxiety, and as the increasing anxiety triggers dyspnea again, the patient enters a cycle.

Increased dyspnea due to anxiety often triggers rapid shallow breathing, which in turn leads to increased dead space ventilation, reduced alveolar ventilation, and worsened gas exchange. Breaking this vicious cycle with behavioral or pharmacological symptom management can improve physiological status while reducing suffering. Determining the severity of dyspnea, anxiety and death anxiety levels in COPD patients is important for planning applications for these problems and implementing individual care (Banzett et al. 2020). Dyspnea causes them to experience physical limitation, fatigue, limited activities of daily living, elevated level of dependency, fear of death, stress, anxiety, and depression. It is important to evaluate dyspnea in detail in patients diagnosed with COPD and to apply treatments for them accordingly (Kapısız and Eker 2018). The palliative care approach, which provides holistic care including the patient, their caregiver, or close relatives, is crucial for individuals with COPD (WHO 2022). In COPD, which progresses slowly and has an irreversible course, symptoms such as dyspnea, anxiety, and fear of death become more intense as death approaches. Since this situation highlights the need for palliative care, it is important to plan appropriate palliative care (Gürsan and Erdoğan 2022).

Nurses play a key role in the treatment and management of COPD since they are constantly in contact with patients. Nursing care in COPD focuses on managing symptoms of patients, developing their self-care skills, and improving their functions (Bilgehan et al. 2020).

In the care of patients with COPD, it is important to evaluate their psychological status in addition to physical care and medical treatment. Nurses will primarily define existing problems and create a database for the studies to be conducted in this way. Therefore, the aim of this study is to identify levels of dyspnea, death anxiety, and anxiety in patients with COPD. It is thought that results of the present study would be guiding for nursing care and practices and would contribute to the future related studies.

### Objectives

The studies conducted with COPD patients in Türkiye have mainly focused on self-care agency (Ergin et al. 2022), quality of life (Karagülle and Can Çiçek 2020), and fatigue (Saza and Çevik 2020). Moreover, there are a limited number of studies in the literature examining the relationship between dyspnea, anxiety, and depression (Kapısız and Eker 2018) and the relationship between anxiety and death anxiety (Nal et al. 2016). No study was found in the literature evaluating dyspnea, death anxiety, and anxiety together in individuals with COPD. The aim of the study is to fill the gap in the literature on this subject, to determine the levels of dyspnea, anxiety, and death anxiety in patients with COPD and the relationship between them, and to guide evidence-based practices on this subject.

### Research Questions

- What is the level of dyspnea in patients with COPD?
- What is the level of anxiety in patients with COPD?
- What is the level of death anxiety in patients with COPD?
- What are the factors affecting dyspnea, anxiety, and death fear in patients with COPD?
- Is there any correlation between levels of dyspnea, death anxiety, and anxiety in patients with COPD?

### Hypotheses of the Study

- As the severity of dyspnea increases in individuals with COPD, the level of anxiety also increases.
- As the severity of dyspnea increases in individuals with COPD, the level of death anxiety also increases
- There is a relationship between the severity of dyspnea, anxiety, and death anxiety levels in individuals with COPD.

## Methods

### Participants

The sample size of the study was calculated to be 199 at an effect size of d = 0.20, a margin of error of 5%, and power of 80% after power analysis done in the G*Power 3.0.10 program. The study was conducted with 200 patients who applied to the Chest Diseases Outpatient Clinic with the diagnosis of COPD, met the inclusion criteria, and agreed to participate in the study.

### Study design and setting

This descriptive study was conducted in the chest diseases outpatient clinic of a state hospital in Türkiye between 01.12.2022 and 30.06.2023.

**Inclusion Criteria**

- Being aged 18 years and older.
- Having no vision and hearing impairments.
- Having been diagnosed with COPD at least 6 months before.
- Having no cognitive dysfunction.
- Having no communication problems.
- Having no diagnosis of psychiatric disorders.
- Being volunteered to participate in the study.

### Data collection tools

A Patient Information Form, the Death Anxiety Scale, the Beck Anxiety Inventory, the Modified British Medical Research Council (mMRC) Dyspnea scale, and the Modified Borg Scale were used to collect data. While the Borg scale allows the evaluation of dyspnea severity during exercise and rest, the mMRC scale allows the natural course of the disease to be followed (Kara and Yıldız 2013). In this respect, the use of these two dyspnea scales was found appropriate.

### Patient information form

A patient information form was prepared by the researchers upon the literature review (Nal et al. 2016). It consists of 19 questions about the socio-demographic and disease-related characteristics of the COPD patients.

### Death anxiety scale (DAS)

The Death Anxiety Scale was developed by Templer in 1970 and was adapted into Turkish by Şenol in 1989 (Şenol 1989; Templer 1970). This two-point Likert-type scale consists of 15 items, which are rated as true or false. The score of the scale ranges between 0 and 15. The highest score of the scale is 15. Getting a score between 0 and 4 points indicates “mild level of death anxiety,” getting a score between 5 and 9 points indicates “moderate level of death anxiety,” getting a score between 10 and 14 points indicate “severe level of death anxiety,” and 15 points indicate “panic level” of death anxiety”. In the study by Şenol, the Cronbach’s alpha coefficient of the scale was found to be 0.86 (Şenol 1989). In the present study, the reliability coefficient of the scale was found to be 0.875.

### Beck anxiety inventory (BAI)

The inventory was developed by Beck et al. (1988) to assess the frequency of anxiety symptoms. Ulusoy et al. conducted its Turkish validity and reliability study in 1998. This 4-point Likert-type scale consists of 21 items. The score of the scale ranges from 0 to 63. A score between 8 and 15 points on the scale indicates mild anxiety, a score between 16 and –25 points indicates moderate anxiety; and 26 and above points indicate severe anxiety. In their study, Ulusoy et al. calculated the Cronbach’s alpha coefficient of the scale as 0.93 (Ulusoy et al. 1998). In the present study, its Cronbach’s alpha coefficient was found to be 0.814.

### Modified British Medical Research Council (mMRC) dyspnea scale

This is a five-item Likert-type scale based on various physical activities that cause dyspnea. The score of this scale ranges from 0 to 4, and the patients select the most appropriate option that best describes their respiratory distress (Bestall et al. 1999). Fletcher first used this scale in the 1940s to compare people with and without pulmonary disease in terms of the severity of dyspnea they experienced during activities (Fletcher et al. 1959). Later, the British Medical Research Council modified this scale to monitor the natural course of the disease (Bestall et al. 1999). Studies that compared dyspnea scales in Türkiye reported that the scale can be safely used to assess dyspnea (Yapucu Günes et al. 2012).

### Modified Borg Scale (MBS)

The Modified Borg Scale was developed by Borg in 1970 to measure the level of effort people make during physical activity. Today, this unidimensional scale is generally used to assess the severity of exertional dyspnea and the severity of resting dyspnea. It consists of 10 items that describe the severity of dyspnea according to their degree. The scoring ranges from 0 (none) to 10 (highly severe) (Borg 1982).

### Data analysis

Statistical analyses were performed using the SPSS (IBM SPSS Statistics 27) packaged software. Frequency tables and descriptive statistics were used to interpret the findings.

For non-normally distributed data, non-parametric tests were run. The Mann-Whitney U test (Z-table value) was used to compare the data of two independent groups, while the Kruskal-Wallis H test (χ^2^-table value) was utilized to compare the data of three or more independent groups. For the two-group comparisons of variables that caused a difference in three-group comparisons, Bonferroni correction was implemented. The “Spearman” correlation coefficient was used to analyze the correlation between non-parametric values. The significance level was set at p<0.05.

### Ethical considerations

Ethical approval from the Non-Interventional Clinical Trials Ethics Committee of a university’s Faculty of Medicine (04.11.2022/50) and institutional permission from the related hospital was obtained to conduct the study. Moreover, verbal and written informed consent was obtained from the participants.

### Results

Results of the present study revealed that the mean age of the patients was 66.73 ± 8.45 years and 50.5% of them were in the age group of 61–70 years. A total of 73.0% of the patients were male, 78.5% were married, 59.0% were primary school graduate, 56.0% were retired, 58.0% had a low income, 75.0% were living in the province, and 44.5% were living with their spouses. In all, 59.5% of the patients had quit smoking, 71.0% were independent in activities of daily living, and 67.5% perceived their health as poor (Table 1).

**Table 1.** Descriptive characteristics of the patients

Characteristics (N = 200)	n	%
Age group [ 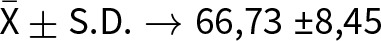 ]		
≤60	40	20.0
61 − 70	101	50.5
>70	59	29.5
**Gender**		
Female	54	27.0
Male	146	73.0
**Marital status**		
Married	157	78.5
Single	43	21.5
**Educational level**		
Illiterate/literate	43	21.5
Primary school	118	59.0
High school/Bachelor’s degree	39	19.5
**Employment status**		
Retired	112	56.0
Housewife	43	21.5
Other	45	22.5
**Income level**		
High	16	8.0
Moderate	68	34.0
Low	116	58.0
**Place of residence**		
Village	16	8.0
District	34	17.0
Province	150	75.0
**Person(s) sharing living quarters**		
Alone	29	14.5
With children	28	14.0
With spouse	89	44.5
With spouse and children	54	27.0
**Smoking status**		
No	23	11.5
Quitted	119	59.5
Yes	58	29.0
**Duration of smoking**		
40 years and less	91	51.4
40 years and more	86	48.6
**Number of cigarettes they smoke (pack/day)**		
Less than 1	29	16.4
1	106	59.9
2 − 3	42	23.7
**Dependence status**		
Dependent	6	3.0
Semi-dependent	52	26.0
Independent	142	71.0
**Perception of health**		
Good	14	7.0
Moderate	51	25.5
Poor	135	67.5

When the disease-related characteristics of the participants were analyzed, it was found that 39.0% of them were diagnosed for 10 years or more, 37.5% suffered from severe stage COPD, 60.0% had comorbid chronic diseases, and 86.0% were on regular medication. In all, 26.0% were continuously on oxygen therapy, 68.0% did not attend regularly follow-up visits, 42.5% were hospitalized 1–2 times in the last year, and 35.5% experienced exacerbations 2–3 times in the last year (Table 2).

**Table 2.** Disease-related characteristics of the patients

Characteristics (N = 200)	n	%
Duration of diagnosis		
≤1 year	22	11.0
2 − 5 years	59	29.5
6 − 9 years	41	20.5
≥10 years	78	39.0
**Stage of COPD**		
Mild	8	4.0
Moderate	59	29.5
Severe	75	37.5
Very severe	58	29.0
**Presence of comorbid chronic diseases**		
Yes	120	60.0
No	80	40.0
**Status of being on regular medication**		
Yes	172	86.0
No	28	14.0
**Status of being on oxygen therapy**		
Yes	52	26.0
No	148	74.0
**Status of receiving oxygen**		
<15 hours	30	57.7
≥15 hours	22	42.3
**Status of attending regularly follow-up visits**		
Yes	64	32.0
No	136	68.0
Frequency of attending follow-ups (n = 64)*		
Every two months	19	29.7
Every three months	21	32.8
Monthly	24	37.5
**Hospitalization in the last year**		
None	72	36.0
1 − 2 times	85	42.5
3 and more times	43	21.5
**Number of exacerbations in the last year**		
1 time	29	14.5
2 − 3 times	71	35.5
4 − 5 times	68	34.0
>5 times	32	16.0

The mean scores of the participants were 5.21 ± 2.46 on the MBS, 2.62 ± 1.03 on the mMRC, 17.87 ± 7.96 on the BAI, and 10.07 ± 4.02 on the DAS. While 40.5% of the patients had mild or moderate levels of anxiety, 41.5% had severe level of death anxiety (Table 3).

**Table 3.** Distribution of the patients’ MBS, mMRC, BAI, and DAS mean scores

Scale (N = 200)	*X* ± S.D.	Median (Min-Max)
**MBS**	5.21 ± 2.46	5.0(1.0 − 10.0)
**mMRC**	2.62 ± 1.03	3.0(1.0 − 4.0)
**BAI**	17.87 ± 7.96	17.0(3.0 − 52.0)
**DAS**	10.07 ± 4.02	11.0(1.0 − 15.0)
	**n**	**%**
**BAI Levels**		
Mild	81	40.5
Moderate	81	40.5
High	38	19.0
**DAS Levels**		
Mild	24	12.0
Moderate	61	30.5
Severe	83	41.5
Panic	32	16.0

MBS; Modified Borg Scale, mMRC; Modified British Medical Research Council Dyspnea Scale, BAI; Beck Anxiety Inventory, DAS; Death Anxiety Scale.

When the MBS and mMRC scores of the patients were analyzed, it was observed that the dyspnea scores of the patients who were in the age group of 70 years and older, male, single, retired, had a low income, were living with their children, smoked for more than 40 years, were dependent in activities of daily living, had a very severe disease stage, had comorbid chronic diseases, were on regular medication, perceived their health as poor, were on oxygen therapy, received oxygen for 15 hours or more, attended regularly follow-up visits, were hospitalized 3 or more times, and experienced exacerbations more than five times per year were higher than those of the patients in the other group. Moreover, the MBS scores of the patients who were diagnosed for 6–9 years and the mMRC scores of those who were diagnosed for 10 years or more were higher than those of the patients in the other groups (*p* < 0.05).

When BAI scores of the patients were analyzed, it was found that the patients who were in the age group of 61–70 years, female, single, housewives, smoked for more than 40 years, were semi-dependent in activities of daily living, diagnosed for 6–9 years, suffered from COPD at a very severe stage, had a comorbid chronic disease, were on regular medication, perceived their health as poor, were on oxygen therapy, received oxygen for 15 hours or more, attended regularly follow-up visits, were hospitalized 3 or more times, and experienced exacerbations more than five times per year had higher BAI scores (*p* < 0.05).

When DAS scores of the patients with COPD were examined, it was found that those who were female, housewife, had comorbid chronic diseases, and received oxygen for 15 hours or more had higher DAS scores and this difference was statistically significant (*p* < 0.05).

There was a positive, very high, and statistically significant correlation between mMRC and MBS scores of the patients (r = 0.867, *p* < 0.001). BAI had a positive, weak, and statistically significant correlation with mMRC and MBS (r = 0.419, *p* < 0.001; r = 0.377, *p* < 0.001). Furthermore, there was a positive, very weak/moderate, and statistically significant correlation between the patients’ DAS, mMRC, MBS, and BAI scores (r = 0.156, *p* = 0.027; r = 0.243, *p* < 0.001; r = 0.506, *p* < 0.001) (Table 4).

**Table 4.** Correlation between the patients’ scale scores

Scales		MBS	mMRC	BAI	DAS
**MBS**	*r*	1.000	0.867	0.377	0.236
	*p*	-	**<0.001**	**<0.001**	**<0.001**
**mMRC**	*r*		1.000	0.419	0.156
	*p*		-	**<0.001**	**0.027**
**BAI**	*r*			1.000	0.506
	*p*			-	**<0.001**
**DAS**	*r*				1.000
	*p*				-

MBS; Modified Borg Scale, mMRC; Modified British Medical Research Council Dyspnea Scale, BAI; Beck Anxiety Inventory, DAS; Death Anxiety Scale.

## Discussion

Dyspnea is a common distressing and debilitating symptom in patients with COPD (Hanania and Donnell 2019). The present study revealed that the mMRC mean score of the participants was 2.62 ± 1.03. The GOLD 2023 COPD Guideline considers 2 points and above as moderate-to-severe dyspnea (GOLD 2023). Findings of the present study indicated that the dyspnea level of the participants was moderate-severe. Similarly, a study by Kapısız and Eker (2018) reported that the patients with COPD perceived their dyspnea as severe (MRCS: 3.1 ± 1.37) (Kapısız and Eker 2018). In their study, Özdemir and Enç (2022) found that the mMRC mean score was 3.23 ± 1.29 (Özdemir and Enç 2022). The present study revealed that the patients had a mean score of 5.21 ± 2.46 on the MBS. The study by Yapucu Günes et al. (2012) reported that the MBS mean score was 4.20 ± 2.10 (Yapucu Günes et al. 2012). In their study, Sengupta et al. (2020) found that the MBS mean score of COPD patients who applied to the emergency department was 5.18 ± 2.33. The optimum threshold in Borg score is 4; a score below 4 points signifies safe discharge (Sengupta et al. 2020). In the present study, it was determined that the patients had high levels of dyspnea, which was associated with the fact that most of the patients were in the severe stage of COPD.

Anxiety disorders are among the most prevalent psychiatric comorbidities in patients with COPD (Christiansen et al. 2023). In the present study, it was found that 40.5% of the patients with COPD had moderate level of anxiety. Likewise, the study by Korkmaz and Tel (2010) reported that 43.7% of the patients experienced moderate anxiety (Korkmaz and Tel 2010). A study conducted by Thapa et al. (2017) with patients with COPD indicated that 41.9% of them had moderate level of anxiety (Thapa et al. 2017). Results of the present study are compatible with the literature.

Death anxiety is quite common in patients with COPD due to dyspnea, dependence on hospitalization, continuous drug use, and dysfunction of the affected organs (Taytard and Cousson 1996). Almost half (41.5%) of the patients in the present study were found to have severe level of death anxiety. Unlike the present study, a review of studies conducted with patients with COPD showed that these patients had moderate level of death anxiety (Nal et al. 2016; Togluk and Çuhadar 2021). In the present study, it was found that almost half of the patients had severe level of death anxiety since their age range was 61–70 years and they reached severe stage of COPD and had dyspnea, and high levels of anxiety.

Cultural differences and religious beliefs are significant factors in shaping individuals’ attitudes toward death anxiety (Semerci Çakmak et al. 2025). Among Muslims, the belief in the afterlife is an important factor influencing death anxiety, and it is believed that this anxiety arises not from concerns about the end of life but from the feeling of being unprepared for the afterlife (Gürbüz and Yorulmaz 2024). A study conducted in Pakistan reported that Muslims experience higher levels of death anxiety (Husain et al. 2024). Along with our study in Turkey, studies conducted in Tehran (Majidi et al. 2019) and the Turkish Republic of Northern Cyprus (Çayırcı et al. 2024) have similarly shown that individuals with COPD have high levels of death anxiety. Understanding and respecting the cultural differences, beliefs, and traditions of patients and their relatives is crucial for maintaining the integrity of the relationship in palliative care (Wiener et al. 2013).

Dyspnea is a major symptom leading to anxiety and death anxiety. As in all chronic diseases, continuous drug use, dependence on hospitalization, and dysfunction of the affected organs in COPD exacerbate the anxiety of patients towards the future, resulting in hopelessness (Togluk and Çuhadar 2021). In this study, it was found that as the dyspnea levels of patients with COPD elevated, so did their anxiety and death anxiety levels. In the literature, it has been determined that death anxiety intensifies as dyspnea severity increases in COPD patients (Bülbüloğlu and Kaplan Serin 2023). A similar study reported that anxious patients with COPD had more death anxiety (Nal et al. 2016). It was also found that the anxiety level of COPD patients with dyspnea was higher, and their anxiety level elevated with higher dyspnea level (Kapısız and Eker 2018). This shows that dyspnea affects death anxiety and anxiety, and an elevated level of dyspnea leads to fear of drowning and exacerbates death anxiety and anxiety.

## Conclusion

Based on the results of this study, it is recommended that dyspnea, anxiety, and death anxiety levels of patients be periodically assessed, that patients at risk for anxiety and death anxiety be provided follow-up, treatment and care by the Consultation Liaison Psychiatry Department and evidence-based studies be planned to alleviate dyspnea, anxiety, and death anxiety in these patients.

### Limitations

The results of this study can only be generalized to patients with COPD who apply to the chest diseases outpatient clinic of the related hospital where the study was conducted.

### Implications for nursing practice

Nurses play an important role in managing the symptoms in patients with COPD, reducing hospitalizations, and increasing exercise capacity. The goal of palliative care is to ensure the patient’s physical and emotional comfort through symptom-focused treatments. In the management of dyspnea, one of the most common symptoms experienced by patients with COPD, strategies include proper positioning, breathing exercises, airway clearance techniques, ventilatory support, and pulmonary rehabilitation. In addition to these strategies, anxiety management is also of great importance. In this context, nurses should provide a calm and quiet environment for patients, avoid leaving them alone, and ensure psychological or psychiatric support is provided when necessary. From this point of view, nursing care and practices hold great importance in enhancing the quality of life of patients with COPD. It is thought that results of the present study would be guiding for nursing care and practices and contribute to the future studies.

## Data Availability

This study’s data and findings are available from the corresponding author upon a reasonable request.
